# Validation of the HCM Risk-SCD Model in a Chinese Hypertrophic Cardiomyopathy Cohort

**DOI:** 10.3390/jcm14207355

**Published:** 2025-10-17

**Authors:** Fei Hang, Chaomei Fan

**Affiliations:** Department of Cardiology, Fuwai Hospital, National Center for Cardiovascular Diseases, Chinese Academy of Medical Sciences and Peking Union Medical College, Beijing 100037, China; hangfei1936@163.com

**Keywords:** hypertrophic cardiomyopathy, sudden cardiac death, risk stratification, Chinese population

## Abstract

**Background**: Hypertrophic cardiomyopathy (HCM) is associated with sudden cardiac death (SCD). The HCM Risk-SCD model has been widely used in Western populations, but its performance in Chinese patients remains unclear. **Methods**: This retrospective cohort study evaluated 534 HCM patients (348 males and 186 females) at Fuwai Hospital from 1992 to 2010. We calculated the HCM Risk-SCD score for each patient and categorized them into low-risk (<4%) and intermediate–high-risk (≥4%) groups. The primary endpoint was SCD events, defined as unexpected sudden death within one hour of symptom onset, successful resuscitation after cardiac arrest, appropriate ICD discharge, or sustained ventricular tachycardia. Model performance was assessed using Cox regression analysis, Kaplan–Meier survival analysis, ROC curve analysis, and subgroup analyses with interaction tests. **Results**: During a mean follow-up of 6.96 ± 4.16 years, 31 SCD events occurred. The intermediate–high-risk group had significantly higher SCD incidence than the low-risk group (8.68% vs. 3.42%, *p* = 0.01). This association remained significant after multivariate adjustment (HR 2.718, 95% CI: 1.264–5.848, *p* = 0.011). Kaplan–Meier analysis showed significant differences in SCD-free survival between risk strata (log-rank *p* = 0.01). The actual 5-year SCD event rate (4.31%) closely aligned with the model-predicted rate (4.65 ± 3.26%). ROC analysis demonstrated moderate discriminative ability in the overall population (AUC = 0.660, *p* = 0.003). The optimal cutoff value was 3.23 for the overall population. **Conclusions**: The HCM Risk-SCD model demonstrates acceptable performance in Chinese HCM patients.

## 1. Background

Hypertrophic cardiomyopathy (HCM) is the most prevalent inherited cardiovascular disorder, with an estimated global prevalence of 1:500 in the general population [1]. To date, HCM cases have been reported across 122 countries, affecting approximately 20 million individuals worldwide [2]. A multicenter epidemiological study conducted in nine Chinese provinces demonstrated that China had at least 1 million HCM patients in 2003, with an age- and sex-adjusted prevalence rate of 80 per 100,000 population [3]. Characterized by asymmetric myocardial hypertrophy and remarkable genotypic/phenotypic heterogeneity [1], HCM manifests as thickening of the left and/or right ventricular walls, a reduced ventricular chamber size, impaired left ventricular diastolic filling, and decreased diastolic compliance [1,4]. Although most patients exhibit relatively favorable prognoses, the disease may lead to severe complications, including sudden cardiac death (SCD), congestive heart failure, atrial fibrillation, and ventricular arrhythmias. Notably, some patients progress to end-stage dilated cardiomyopathy, exacerbating heart failure symptoms.

SCD is undoubtedly the most severe complication of HCM. The annual incidence of SCD in HCM patients can reach 0.5–1%, and HCM-related SCD remains one of the leading causes of sudden death in young individuals [1,5]. The primary mechanism underlying SCD in HCM is malignant ventricular arrhythmias triggered by myocardial fibrosis, involving electrophysiological abnormalities due to ion channel dysfunction, including remodeling of sodium, potassium, and calcium channels, leading to action potential prolongation and early afterdepolarizations [6]. Concurrently, altered mitochondrial dynamics and excitation–contraction coupling mechanisms resulting in inefficient energy utilization and a blunted Frank–Starling mechanism collectively form the critical pathophysiological substrate promoting arrhythmogenesis [7,8]. Consequently, an implantable cardioverter–defibrillator (ICD) is highly effective in preventing SCD in this population [9,10]. However, ICD implantation may also introduce new complications, including device-related infections (at the implantation site or intracardiac), procedural complications, and inappropriate ICD shocks [11].

Due to the prognostic heterogeneity in HCM, accurate SCD risk stratification is clinically essential to guide targeted interventions for high-risk patients while avoiding unnecessary burdens and resource utilization in low-risk cases. The 2011 American College of Cardiology Foundation/American Heart Association (ACCF/AHA) Guidelines for the Diagnosis and Treatment of HCM established a risk stratification system for SCD in HCM patients, identifying five major risk factors: (1) extreme left ventricular hypertrophy (maximal wall thickness ≥ 30 mm); (2) family history of SCD in first-degree relatives before age 40; (3) unexplained syncope; (4) non-sustained ventricular tachycardia (NSVT) on ambulatory ECG monitoring; and (5) abnormal blood pressure response during exercise (ABPR) [5]. Patients meeting any one of the first three criteria or any two of the five factors were classified as high-risk and recommended for ICD implantation (Class IIa, Level of Evidence C) for SCD prevention [5]. However, the predictive accuracy of this risk stratification model has been increasingly challenged in recent years. A large-scale validation study published in 2013 demonstrated relatively low accuracy in predicting SCD events, suggesting its limited suitability for HCM patient screening [12].

The following year, O’Mahony et al. [13] developed a novel SCD risk stratification model (HCM Risk-SCD) based on epidemiological data from 3675 HCM patients. This mathematical model calculates the 5-year SCD risk, categorizing patients into three tiers: (1) low risk (<4%), (2) intermediate risk (4–6%), and (3) high risk (>6%). Subsequently adopted by the 2014 ESC Guidelines for the Diagnosis and Management of HCM [1], this model recommends ICD implantation for high-risk patients (>6%), considers it for intermediate-risk cases (4–6%), and discourages its use in low-risk patients (<4%) [13]. It is noteworthy that the 2023 ESC Guidelines for the management of cardiomyopathies continue to endorse the HCM Risk-SCD calculator as the primary risk stratification tool (Class I, Level B). However, the guidelines now formally recognize additional risk modifiers that can influence ICD implantation decisions. For patients with low risk (<4%), the presence of extensive late gadolinium enhancement (LGE ≥ 15% of LV mass) or LVEF < 50% may warrant ICD consideration (Class IIb, Level B) [14]. The 2024 AHA/ACC Guidelines further emphasize this individualized approach, incorporating left ventricular apical aneurysm as a major risk factor and highlighting the importance of shared decision-making in ICD implantation [15]. These evolving guidelines underscore the importance of population-specific validation studies to optimize risk prediction across different ethnic groups. The HCM Risk-SCD model has garnered significant academic attention since its introduction, prompting numerous validation studies in Western populations.

While most investigations have supported its predictive validity [16,17,18], some researchers have raised concerns regarding its accuracy [19,20]. Important validation studies have also emerged from Asian populations. In Korea, Choi et al. [21] evaluated the model in 730 HCM patients, reporting an AUC of 0.718, though notably, 63.6% of SCD events occurred in patients classified as low-risk. Similarly, in Japan, Nakano et al. [22] found that only 36% of high-risk patients met the ESC Guideline criteria for ICD implantation, suggesting potential threshold differences in Asian populations.

Recent Chinese validation studies have revealed concerning performance issues with the HCM Risk-SCD model. Liu et al. evaluated 1369 patients at Fuwai Hospital and found that the ESC model had remarkably poor sensitivity of only 12.8%, correctly identifying just 5 of 39 patients who experienced SCD events, while the enhanced ACC/AHA strategy achieved 66.7% sensitivity [23]. Dong et al. validated the 2020 AHA/ACC guidelines in 511 patients from Nanjing, demonstrating superior performance (AUC 0.71) compared to the ESC model (AUC 0.68), with the ESC model showing particularly poor sensitivity at the recommended thresholds [24]. Qi et al. reported even poorer ESC model performance in 856 patients from West China Hospital, with a C-statistic of only 0.58 and correct classification of merely 29.6% of SCD events [25]. Most recently, Ma et al. analyzed 2781 HCM patients from 13 tertiary hospitals, reporting a 4.6% SCD rate and demonstrating significant age and sex differences in risk, though they did not directly validate the HCM Risk-SCD model [26].

While these studies provide valuable insights, they are predominantly single-center investigations with heterogeneous methodologies, follow-up durations, and regional characteristics. Given the vast geographic, economic, and healthcare resource variations across China, additional validation studies from different regions and institutions remain essential to comprehensively assess the model’s applicability in diverse Chinese populations. Furthermore, the consistent pattern of poor ESC model performance across these studies highlights the urgent need for the optimization of risk thresholds or the development of population-specific models for Chinese HCM patients.

## 2. Methods

### 2.1. Study Design and Study Population

This retrospective cohort study was conducted at Fuwai Hospital, Chinese Academy of Medical Sciences. We initially screened 592 consecutive patients with HCM treated between 1 January 1992 and 31 December 2010. Inclusion criteria required the following: (1) echocardiographic confirmation meeting the 2014 ESC HCM diagnostic guidelines; (2) availability of complete clinical data; and (3) no history of SCD events, cardiopulmonary resuscitation, end-stage heart failure, or appropriate ICD therapy. Following the exclusion of 58 patients who were lost to follow-up after exhaustive contact attempts via telephone, medical record review, and social media outreach, the study population for analysis included 534 patients, with 346 males and 186 females. The study protocol was approved by the institutional ethics committee and conducted in accordance with the Declaration of Helsinki.

### 2.2. Clinical Data Collection

#### 2.2.1. General Data Collection

This study comprehensively collected clinical data through a review of hospital electronic medical records and direct inquiries to patients or their families. Specific clinical characteristics included the following: age at evaluation; sex; family history of SCD in first-degree relatives under 40 years of age; history of unexplained syncope, non-sustained ventricular tachycardia (NSVT); paroxysmal/persistent atrial fibrillation (AF), hypertension, and diabetes; New York Heart Association (NYHA) functional classification; and baseline medication use (including non-dihydropyridine calcium channel blockers [CCBs] and β-blockers). Exclusion criteria included the following: (1) patients who had undergone septal reduction therapy (alcohol septal ablation or surgical myectomy) prior to baseline evaluation; (2) patients with other concomitant cardiomyopathies; (3) incomplete clinical data necessary for HCM Risk-SCD calculation.

#### 2.2.2. Echocardiography Examination

Two-dimensional echocardiography was used to assess ventricular wall hypertrophy from parasternal long-axis, short-axis, and apical four-chamber views of the left ventricle. Continuous wave Doppler ultrasound was employed to detect high-velocity abnormal blood flow signals to determine the presence of left ventricular outflow tract obstruction (LVOTO), with color Doppler flow imaging further confirming the obstruction and its location. LVOTO was defined as a pressure gradient ≥ 30 mmHg at rest or after exertion.

Echocardiographic measurements included the following: maximal left ventricular wall thickness (MLVWT); left ventricular outflow tract (LVOT) pressure gradient (at rest or after provocation); left atrial (LA) diameter; left ventricular ejection fraction (LVEF); and left ventricular end-diastolic diameter (LVEDD).

#### 2.2.3. Assessment of HCM Risk-SCD Model

This study estimated the risk of SCD events in HCM patients within 5 years based on the HCM Risk-SCD model formula:

P_SCD at 5 years_ = 1 − 0.998exp^(Prognostic Index)^, where Prognostic Index = 0.15939858 × maximal LVWT (mm) − 0.00294271 × maximal LVWT^2^ (mm^2^) + 0.0259082 × LA diameter (mm) + 0.00446131 × maximal LVOT gradient (mmHg) + 0.4583082 × family history of SCD + 0.82639195 × NSVT + 0.71650361 × unexplained syncope-0.01799934 × age at evaluation (years).

According to the 2014 ESC Guidelines of HCM, HCM patients with an HCM Risk-SCD ≥ 6% were defined as the high-risk group, HCM patients with an HCM Risk-SCD between 4% and 6% were defined as the moderate-risk group, and HCM patients with an HCM Risk-SCD < 4% were defined as the low-risk group. Patients in the moderate- and high-risk groups were recommended for ICD implantation. For the purpose of this study, we combined the moderate- and high-risk groups into an “intermediate–high-risk group” (HCM Risk-SCD ≥ 4%) and compared outcomes with the low-risk group (HCM Risk-SCD < 4%), as both moderate- and high-risk patients are recommended for ICD implantation according to guidelines.

For this study, we combined intermediate- and high-risk groups into an “intermediate–high-risk group” (≥4%) (both groups receive similar ICD implantation recommendations in clinical practice (Class IIa–IIb); the limited number of high-risk patients (≥6%, n = 89, 16.7%) would have insufficient statistical power for three-group comparisons). This approach has been used in previous validation studies to enhance statistical reliability.

### 2.3. Follow-Up and Endpoint Definition

We followed HCM patients evaluated between 1 January 1992 and 31 December 2010. In our study, a subset of patients was followed up via secure social media platforms in addition to standard clinical visits or telephone contacts. Patients provided updates on their health status through these platforms, which were then verified by trained study personnel through direct messaging or video calls. All communications adhered to privacy regulations, and any reported events of interest, including SCD or hospitalizations, were confirmed via medical records whenever possible. Patients were considered lost to follow-up if no contact could be established after at least three consecutive attempts using different methods of communication over a period of 3 months.

The primary endpoint of this study was SCD events, which were defined as a composite of the following occurrences: (1) unexpected sudden death occurring within one hour of symptom onset; (2) successful cardiopulmonary resuscitation after cardiac arrest; (3) appropriate ICD discharge for ventricular fibrillation or persistent rapid ventricular tachycardia (>200 beats/min); (4) sustained rapid ventricular tachycardia (>200 beats/min) affecting hemodynamic stability and leading to cardioversion. The occurrence of primary endpoint events and their timing were meticulously recorded throughout the follow-up period.

### 2.4. Statistical Analysis

Categorical variables were expressed as frequencies and percentages. The normality of continuous variables was assessed using the Shapiro–Wilk test. Normally distributed continuous variables were presented as mean ± standard deviation, while non-normally distributed variables were presented as median (interquartile range [IQR]). For baseline characteristics comparisons between HCM Risk-SCD categories (low-risk vs. intermediate–high-risk), Student’s *t*-test or the Mann–Whitney U test were used for continuous variables, and chi-square tests were used for categorical variables.

To evaluate the association between HCM Risk-SCD categories and SCD events, three Cox regression models were constructed. Model 1 was unadjusted. Model 2 included adjustments for hypertension. Model 3 incorporated additional clinically relevant adjustments for sex and hypertension.

Kaplan–Meier curves were constructed to illustrate the cumulative incidence of SCD events between risk groups, with differences compared using the log-rank test. The actual 5-year SCD event rate was calculated using the Kaplan–Meier method and compared with the mean predicted 5-year SCD event rate estimated by the HCM Risk-SCD model.

Receiver operating characteristic (ROC) curve analysis was performed to evaluate the discriminative performance of the HCM Risk-SCD model in predicting SCD events. The area under the curve (AUC) with 95% confidence intervals (calculated using DeLong’s method for correlated ROC curves) was calculated for the overall population. Youden’s index method (maximum value of sensitivity + specificity − 1) was used to determine the optimal cutoff values of the HCM Risk-SCD score in the overall population.

Subgroup analyses were conducted to assess the consistency of the association between HCM Risk-SCD categories and SCD events across different patient characteristics. Interaction terms were added to the regression models to test for statistical interactions between the HCM Risk-SCD category and subgroup variables, including sex, age (≤47 vs. >47 years, divided by median), hypertension, atrial fibrillation, and NYHA classification (III–IV vs. I–II).

Statistical significance was defined as a two-tailed *p*-value of less than 0.05. All statistical analyses were performed using the R software environment (Version 4.3.2; The R Foundation; available at http://www.R-project.org) (accessed on 3 June 2025).

## 3. Results

This retrospective cohort study initially screened 592 consecutive patients with HCM. After excluding 58 cases lost to follow-up, the final cohort comprised 534 patients for analysis. The study population had a median age of 47.00 years (IQR: 38.00–57.00), with 34.83% (n = 186) being female. Over a mean follow-up duration of 6.96 ± 4.16 years, we observed 31 incident SCD events, corresponding to an annual SCD rate of 0.83%.

### 3.1. Baseline Characteristics

Based on the HCM Risk-SCD score, patients were categorized into low-risk (n = 292, 54.68%) and intermediate–high-risk groups (n = 242, 45.32%). As shown in Table 1, the intermediate–high-risk group exhibited markedly higher HCM Risk-SCD scores than the low-risk group (*p* < 0.001). Compared to low-risk patients, those in the intermediate–high-risk group were significantly younger, had a greater prevalence of family history of SCD, more frequent history of syncope, and higher proportions of NYHA classes III–IV (all *p* < 0.05). Additionally, intermediate–high-risk patients exhibited a lower prevalence of hypertension and diabetes (all *p* < 0.05). Regarding echocardiographic parameters, the intermediate–high-risk group demonstrated greater maximum left ventricular wall thickness, a larger left atrial diameter, and an increased left ventricular outflow tract gradient (all *p* < 0.05). No significant differences were observed between the two groups for other baseline variables (all *p* > 0.05).

### 3.2. SCD Event Distribution

During the follow-up period, significant differences in SCD and related events were observed between risk groups (Table 2). The overall SCD rate was significantly higher in intermediate–high-risk patients (8.68%) compared to low-risk patients (3.42%, *p* = 0.01). This pattern was particularly evident for unexpected sudden death within one hour (6.20% vs. 2.40%, *p* = 0.028). However, other SCD-related events, including successful resuscitation after cardiac arrest (1.65% vs. 1.03%, *p* = 0.707) and appropriate ICD discharges (0.83% vs. 0.00%, *p* = 0.205), did not reach statistical significance.

### 3.3. The Association Between HCM Risk-SCD Categories and SCD

The association between HCM Risk-SCD categories and SCD risk was evaluated through multivariable Cox regression analysis (Table 3). In Model 1 (crude analysis), intermediate–high-risk patients showed a significantly elevated SCD risk compared to low-risk patients (HR 2.599, 95% CI 1.223~5.523, *p* = 0.013). This significant association persisted in Model 2 after adjusting for hypertension (HR 2.699, 95% CI 1.256~5.801, *p* = 0.011) and remained statistically significant in Model 3 following additional adjustments for sex and hypertension (HR 2.718, 95% CI 1.264~5.848, *p* = 0.011).

As shown in Table 4, the calibration analysis demonstrated good agreement between predicted and observed risks, particularly in the intermediate–high-risk group (O/E ratio 0.97, *p* = 0.89). The low-risk group showed slight underestimation (O/E ratio 1.18), though this was not statistically significant (*p* = 0.62).

### 3.4. Survival Analysis

**Figure 1:**
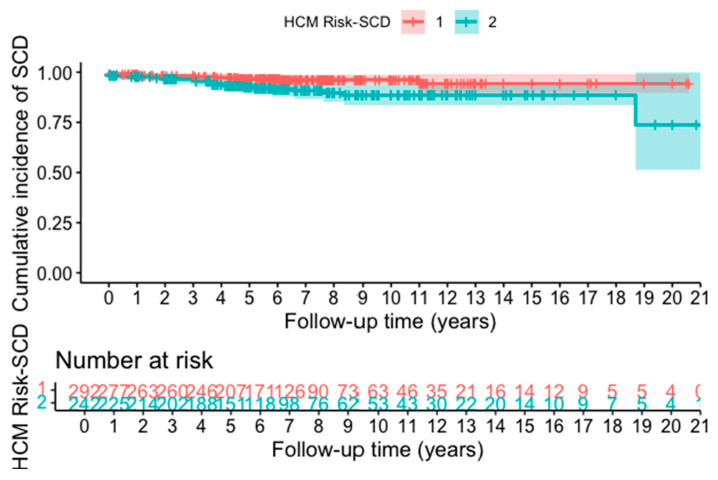
Cumulative incidence of SCD by HCM Risk-SCD categories.

The Kaplan–Meier survival analysis demonstrated highly significant differences in SCD-free survival between risk strata (log-rank *p* = 0.01) (Figure 1). Patients in the intermediate–high-risk group developed substantially higher cumulative SCD incidence compared to low-risk counterparts, with curve separation emerging within the first two years of follow-up and progressively widening thereafter. Notably, the actual 5-year SCD event rate determined by Kaplan–Meier analysis was 4.31%, which closely aligned with the mean 5-year SCD event rate estimated by the HCM Risk-SCD model (4.65 ± 3.26%). Among the 31 SCD events observed during follow-up, the following occurred: unexpected sudden death within one hour: 22 patients (71.0%); successful resuscitation after cardiac arrest: 7 patients (22.6%); appropriate ICD discharge for VF/VT: 2 patients (6.4%); sustained VT with hemodynamic compromise: 0 patients (0%).

### 3.5. ROC Curve Analysis

**Figure 2:**
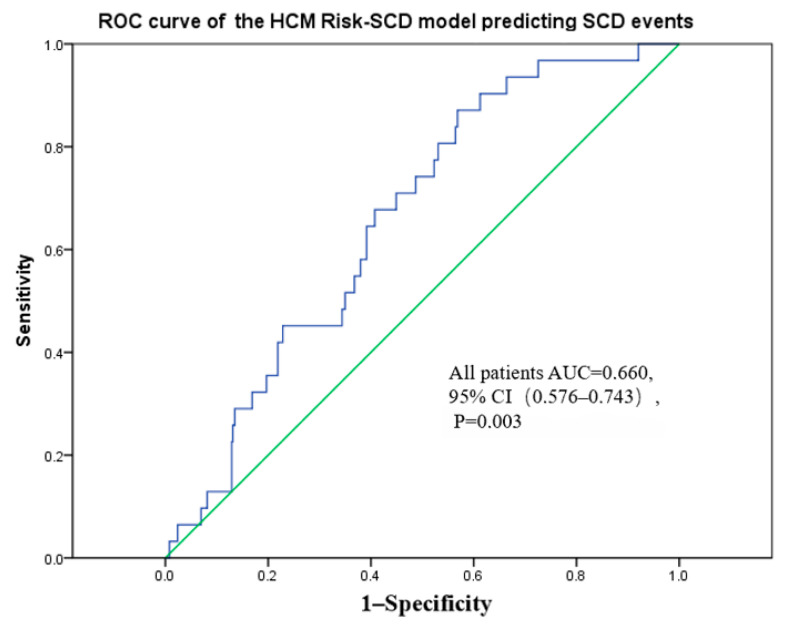
Discriminative performance of HCM Risk-SCD score for predicting SCD in the overall population.

ROC curve analysis was performed to evaluate the discriminative ability of the HCM Risk-SCD score for predicting SCD. In the overall patient population, the HCM Risk-SCD score demonstrated moderate discriminative power with an AUC of 0.660 (95% CI: 0.576–0.743, *p* = 0.003) (Figure 2). At the optimal threshold of 3.23% determined by Youden’s index, the model demonstrated a sensitivity of 87.1% and a specificity of 43.1%.

### 3.6. Subgroup Analysis

Subgroup analyses (Table 5) were conducted based on sex, age (≤47 vs. >47 years), hypertension, atrial fibrillation, and NYHA classifications III–IV. No significant interactions were found for the other subgroups (all *p* for interaction > 0.05).

## 4. Discussion

In this retrospective cohort study, we evaluated the HCM Risk-SCD model’s predictive performance in 534 Chinese HCM patients (348 males and 186 females) with a mean follow-up of 6.96 ± 4.16 years. The model demonstrated moderate discriminative ability in our cohort (AUC = 0.660, 95% CI: 0.576–0.743, *p* = 0.003). Patients stratified as intermediate/high-risk (≥4%) showed significantly higher SCD incidence than low-risk patients (8.68% vs. 3.42%, *p* = 0.010), with a nearly three-fold increased risk after multivariate adjustment (HR 2.718, 95% CI: 1.264–5.848). Furthermore, the optimal threshold value determined by Youden’s index method was 3.23, lower than the 4.0 recommended by European guidelines, suggesting a potential need for threshold adjustment in Chinese populations.

Our findings hold significant implications when compared to previous validation studies of the HCM Risk-SCD model. The overall predictive performance observed in our Chinese cohort (AUC = 0.660) aligns with the AUC value (0.70) reported in the original European cohort, confirming the model’s basic cross-racial applicability [13]. Among Asian populations, Choi et al. [21] demonstrated a higher AUC value (0.718) in 730 Korean patients; however, 63.6% of SCD events occurred in the low-risk group (HCM Risk-SCD < 4%), suggesting that the model may underestimate actual risk in Asian patients. In Japan, Nakano et al. [22] observed a 5-year appropriate discharge rate as high as 50% in the ESC high-risk group with ICD implantation, while only 36% of patients met the Class I/IIa recommendations of ESC Guidelines, further confirming the threshold shift phenomenon in Asian populations.

Chinese validation studies have shown consistently poor ESC model performance. Liu et al. evaluated 1369 patients at Fuwai Hospital, finding that the ESC model had only 12.8% sensitivity versus 66.7% for the enhanced ACC/AHA strategy [23]. Dong et al. reported an AUC of 0.68 for the ESC model versus 0.71 for the 2020 AHA/ACC guideline in 511 patients, with a sensitivity of only 20% at the 4% threshold [24]. Qi et al. found even poorer performance (AUC 0.58) in 856 patients, correctly identifying just 29.6% of SCD events [25]. Ma et al. analyzed 2781 patients from 13 hospitals, demonstrating age as an independent SCD predictor, though without direct model validation [26]. These convergent findings across Asian cohorts, particularly Chinese studies showing AUC values of 0.58–0.68 and sensitivities as low as 12.8–30%, strongly suggest that the European-derived model requires fundamental reconsideration for Asian populations. Our study’s performance falls within this range, reinforcing evidence that the ESC model systematically underperforms in Chinese patients compared to both Western cohorts and the 2020 AHA/ACC guidelines.

The moderate discriminatory capacity (AUC 0.660) suggests opportunities for model improvement. Recent advances indicate that several strategies could enhance risk prediction in Asian populations. First, incorporation of cardiac magnetic resonance (CMR) parameters, particularly late gadolinium enhancement (LGE) quantification, has shown significant incremental value. Chan et al. [27] demonstrated that LGE ≥ 15% of myocardial mass improved the C-statistic by 0.08 in young HCM patients. Second, the integration of novel biomarkers, including high-sensitivity troponin and NT-proBNP, could refine risk stratification. Third, machine learning approaches incorporating multiple imaging and clinical parameters have achieved AUCs exceeding 0.80 in recent studies [28]. The optimal threshold of 3.23% identified in our Chinese cohort has important clinical implications for ICD decision-making. This lower threshold would reclassify approximately 18% (n = 96) of our cohort from low to intermediate risk, potentially identifying an additional 12 patients who experienced SCD events during follow-up. Using this threshold, the Number Needed to Treat (NNT) to prevent one SCD at 5 years would be 14, compared to NNT of 19 using the ESC 4% threshold. Notwithstanding these comparative nuances, our results substantiate the model’s capacity for meaningful risk stratification in the Chinese HCM population, providing clinically actionable prediction of SCD events.

However, lowering the threshold must be balanced against increased ICD-related complications. Contemporary data indicate inappropriate shock rates of 1.6–3.7% annually and device complications in 3.6% per year [11]. Cost-effectiveness analyses suggest that the 4% threshold achieves optimal cost-per-QALY in European populations [26]. Nevertheless, given the high proportion of SCD events occurring in patients classified as low-risk by European thresholds, a population-specific approach may be warranted for Asian patients pending the development of dedicated risk models.

Our study presents several notable strengths. First, it represents one of the largest Chinese HCM cohorts to date (534 patients), providing adequate statistical power. Second, the relatively long follow-up period (mean 6.96 years) enabled us to capture a sufficient number of SCD events (31 cases) for reliable analysis. Third, we identified an optimal risk threshold (3.23) for Chinese HCM patients—a finding with direct clinical value for optimizing ICD implantation decisions. Finally, the consistent performance of our prediction model across all prespecified subgroups (all *p*-interactions > 0.05) demonstrates its pan-stratum applicability in the Chinese hypertrophic cardiomyopathy population.

## 5. Study Limitations

Despite providing important insights, our study has several limitations. First, its retrospective design rather than its prospective nature may affect the accuracy of our findings. Second, the loss to follow-up of 58 patients (9.8%) may impact the external validity of our results. Third, the single-center retrospective design introduces potential selection bias and limits generalizability. As China’s national cardiac referral center, Fuwai Hospital likely receives more severe HCM cases, potentially leading to higher event rates than community populations. However, our diverse geographic patient base partially mitigates this limitation. And the long recruitment period (1992–2010) spans significant changes in HCM diagnosis and management, from evolving diagnostic criteria to improvements in imaging and ICD technology. While this temporal heterogeneity may affect data consistency, it also reflects the real-world evolution of HCM care. Fourth, our ICD implantation rate (8.2%) was lower than contemporary Western rates (15–20%), reflecting historical practice patterns and healthcare resource constraints in China. This could lead to an under detection of arrhythmic events that would have been captured as ICD therapies. However, our data may better represent the natural history of HCM in populations where ICD access remains limited. Patients receiving ICDs likely had compelling clinical indications beyond formal risk scores, potentially introducing selection bias, affecting model performance. Fifth, we did not conduct specialized analyses of specific HCM subtypes (such as apical hypertrophy), which are more common in Asian populations and may possess unique risk characteristics. Sixth, this study only included Chinese HCM patients, and given that HCM patients of different ethnicities show variations in the genetic mutation spectrum, clinical phenotypes, and disease progression patterns, our findings may not be completely applicable to populations of other ethnic backgrounds. Future research should undertake multicenter prospective studies to further validate these findings and consider integrating other clinical factors to improve risk assessment models. Additionally, incorporating cardiac magnetic resonance imaging (CMR), genomic data, and novel biomarkers may enhance predictive accuracy [28,29]. In the long term, developing localized SCD risk prediction models for Chinese populations will have significant clinical value.

## 6. Conclusions

The HCM Risk-SCD model demonstrates acceptable performance in predicting SCD risk in Chinese HCM patients. These findings suggest potential threshold adjustments for Asian populations, which may improve clinical decision-making regarding ICD implantation in Chinese HCM patients.

## Figures and Tables

**Table 1:** Baseline characteristics of HCM patients stratified by HCM Risk-SCD categories.

Variables	Total (n = 534)	Low Risk (n = 292)	Intermediate–High-Risk (n = 242)	*p*
HCM Risk-SCD Risk, M (Q_1_, Q_3_)	3.69 (2.36, 5.99)	2.52 (1.81, 3.21)	6.24 (5.02, 8.30)	<0.001
Female, n (%)	186 (34.83)	109 (37.33)	77 (31.82)	0.183
Age (years), M (Q_1_, Q_3_)	47.00 (38.00, 57.00)	52.00 (41.00, 60.00)	44.00 (36.00, 52.00)	0.028
Family SCD history, n (%)	18 (3.37)	2 (0.68)	16 (6.61)	<0.001
NYHA Classification (III–IV), n (%)	205 (38.39)	101 (34.59)	104 (42.98)	0.047
Syncope, n (%)	207 (38.76)	37 (12.67)	170 (70.24)	<0.001
Previous History				
Coronary heart disease, n (%)	62 (11.61)	41 (14.04)	21 (8.68)	0.054
Atrial fibrillation, n (%)	77 (14.42)	38 (13.01)	39 (16.12)	0.310
Hypertension, n (%)	146 (27.34)	100 (34.25)	46 (19.01)	<0.001
Diabetes, n (%)	26 (4.87)	20 (6.85)	6 (2.48)	0.020
Echocardiogram				
MLVWT (mm), M (Q_1_, Q_3_)	23.00 (19.00, 28.00)	21.00 (18.00, 26.00)	24.00 (21.00, 28.00)	0.016
LA diameter (mm), M (Q_1_, Q_3_)	41.00 (37.00, 46.00)	40.00 (36.00, 44.00)	43.00 (38.75, 48.00)	0.021
LVEF (%), M (Q_1_, Q_3_)	70.00 (64.00, 75.00)	70.00 (63.00, 75.00)	71.00 (65.00, 76.00)	0.523
LVEDD (mm), M (Q_1_, Q_3_)	42.00 (39.00, 47.00)	43.00 (39.00, 47.00)	41.00 (38.00, 47.00)	0.150
LVOT gradient (mm Hg), M (Q_1_, Q_3_)	71.5 (49.75, 100.00)	64.00 (0.00, 88.00)	84.00 (61.00, 112.00)	0.007
Medication history				
Baseline β blockers, n (%)	432 (80.90)	230 (78.77)	202 (83.47)	0.169
Non-dihydropyridine CCBs, n (%)	188 (35.21)	108 (37.00)	80 (33.06)	0.344

First quartile; Q_3_: third quartile; HCM, hypertrophic cardiomyopathy; SCD, sudden cardiac death; MLVWT, maximal left ventricular wall thickness; LA, left atria; LVEF, left ventricular ejection fraction; LVEDD, left ventricular end-diastolic diameter; LVOT, left ventricular outflow tract; CCBs, calcium channel blockers.

**Table 2:** Comparison of outcome events of HCM patients stratified by HCM Risk-SCD categories.

Variables	Total (n = 534)	Low-Risk (n = 292)	Intermediate–High-Risk (n = 242)	*p*
SCD, n (%)	31 (5.81)	10 (3.42)	21 (8.68)	0.010
Unexpected sudden death, n (%)	22 (4.12)	7 (2.40)	15 (6.20)	0.028
Successful resuscitation, n (%)	7 (1.31)	3 (1.03)	4 (1.65)	0.707
Appropriate ICD discharge, n (%)	2 (0.37)	0	2 (0.83)	0.205

ICD, implantable cardioverter–defibrillator.

**Table 3:** Multivariable Cox regression analysis of HCM Risk-SCD score for predicting SCD.

Variables	Model 1		Model 2		Model 3	
	HR (95% CI)	*p*	HR (95% CI)	*p*	HR (95% CI)	*p*
HCM Risk SCD rank						
low risk	1.00 (Reference)		1.00 (Reference)		1.00 (Reference)	
intermediate–high risk	2.599 (1.223~5.523)	0.013	2.699 (1.256~5.801)	0.011	2.718 (1.264~5.848)	0.011

HR: hazard ratio; CI: confidence interval. Model 1: crude; Model 2: adjust: hypertension; Model 3: adjust: hypertension and gender.

**Table 4:** Calibration analysis of the HCM Risk-SCD model by risk category.

Risk Category	N	Mean Predicted Risk (%)	SD	Observed Risk (%)	O	E	O/E Ratio	95% CI	*p*-Value
Low (<4%)	292	2.52	0.83	2.98	8.69	7.36	1.18	0.62–2.25	0.62
Intermediate–high (≥4%)	242	7.22	3.24	6.97	16.87	17.48	0.97	0.60–1.55	0.89

**Table 5:** Subgroup analyses of the association between HCM Risk-SCD categories and SCD risk.

Variables	n (%)	1	2	HR (95% CI)	*p*	*p* for Interaction
All patients	534 (100.00)	10/292	21/242	2.599 (1.223~5.523)	0.013	
Age						0.576
≤47 years old	269 (50.37)	4/116	11/153	2.192 (0.698~6.887)	0.179	
>47 years old	265 (49.63)	6/176	10/89	3.581 (1.301~9.856)	0.014	
>Sex						0.395
male	348 (65.17)	5/183	15/165	3.360 (1.218~9.270)	0.019	
female	186 (34.83)	5/109	6/77	1.719 (0.524~5.633)	0.371	
Hypertension						0.571
no	388 (72.66)	7/192	16/196	2.329 (0.958~5.663)	0.062	
yes	146 (27.34)	3/100	5/46	3.937 (0.940~16.488)	0.061	
NYHA Classification (III–IV)						0.374
no	329 (61.61)	8/191	11/138	2.028 (0.815~5.047)	0.129	
yes	205 (38.39)	2/101	10/104	4.593 (1.006~20.975)	0.049	

HR: hazard ratio; CI: confidence interval.

## Data Availability

The datasets used and/or analyzed during the current study are available from the corresponding author on reasonable request.

## References

[1] Elliott P.M., Anastasakis A., Borger M.A., Borggrefe M., Cecchi F., Charron P., Hagege A.A., Lafont A., Limongelli G., Mahrholdt H. (2014). 2014 ESC Guidelines on diagnosis and management of hypertrophic cardiomyopathy: The Task Force for the Diagnosis and Management of Hypertrophic Cardiomyopathy of the European Society of Cardiology (ESC). Eur. Heart J..

[2] Maron B.J. (2018). Clinical Course and Management of Hypertrophic Cardiomyopathy. N. Engl. J. Med..

[3] Zou Y., Song L., Wang Z., Ma A., Liu T., Gu H., Lu S., Wu P., Zhang Y., Shen L. (2004). Prevalence of idiopathic hypertrophic cardiomyopathy in China: A population-based echocardiographic analysis of 8080 adults. Am. J. Med..

[4] Maron B.J., Maron M.S. (2013). Hypertrophic cardiomyopathy. Lancet.

[5] Gersh B.J., Maron B.J., Bonow R.O., Dearani J.A., Fifer M.A., Link M.S., Naidu S.S., Nishimura R.A., Ommen S.R., Rakowski H. (2011). 2011 ACCF/AHA guideline for the diagnosis and treatment of hypertrophic cardiomyopathy: A report of the American College of Cardiology Foundation/American Heart Association Task Force on Practice Guidelines. J. Thorac. Cardiovasc. Surg..

[6] Zhang K., Wang S., Li X., Cui H., Lai Y. (2025). Mechanism of Ion Channel Impairment in the Occurrence of Arrhythmia in Patients with Hypertrophic Cardiomyopathy. Cardiol. Rev..

[7] Moore J., Ewoldt J., Venturini G., Pereira A.C., Padilha K., Lawton M., Lin W., Goel R., Luptak I., Perissi V. (2023). Multi-Omics Profiling of Hypertrophic Cardiomyopathy Reveals Altered Mechanisms in Mitochondrial Dynamics and Excitation-Contraction Coupling. Int. J. Mol. Sci..

[8] Ušaj M., Moretto L., Månsson A. (2022). Critical Evaluation of Current Hypotheses for the Pathogenesis of Hypertrophic Cardiomyopathy. Int. J. Mol. Sci..

[9] Maron B.J., Spirito P., Ackerman M.J., Casey S.A., Semsarian C., Estes N.A., Shannon K.M., Ashley E.A., Day S.M., Pacileo G. (2013). Prevention of sudden cardiac death with implantable cardioverter-defibrillators in children and adolescents with hypertrophic cardiomyopathy. J. Am. Coll. Cardiol..

[10] O’Mahony C., Lambiase P.D., Quarta G., Cardona M., Calcagnino M., Tsovolas K., Al-Shaikh S., Rahman S.M., Arnous S., Jones S. (2012). The long-term survival and the risks and benefits of implantable cardioverter defibrillators in patients with hypertrophic cardiomyopathy. Heart.

[11] Vriesendorp P.A., Schinkel A.F., Van Cleemput J., Willems R., Jordaens L.J., Theuns D.A., van Slegtenhorst M.A., de Ravel T.J., ten Cate F.J., Michels M. (2013). Implantable cardioverter-defibrillators in hypertrophic cardiomyopathy: Patient outcomes, rate of appropriate and inappropriate interventions, and complications. Am. Heart J..

[12] O’Mahony C., Tome-Esteban M., Lambiase P.D., Pantazis A., Dickie S., McKenna W.J., Elliott P.M. (2013). A validation study of the 2003 American College of Cardiology/European Society of Cardiology and 2011 American College of Cardiology Foundation/American Heart Association risk stratification and treatment algorithms for sudden cardiac death in patients with hypertrophic cardiomyopathy. Heart.

[13] O’Mahony C., Jichi F., Pavlou M., Monserrat L., Anastasakis A., Rapezzi C., Biagini E., Gimeno J.R., Limongelli G., McKenna W.J. (2014). A novel clinical risk prediction model for sudden cardiac death in hypertrophic cardiomyopathy (HCM risk-SCD). Eur. Heart J..

[14] Arbelo E., Protonotarios A., Gimeno J.R., Arbustini E., Barriales-Villa R., Basso C., Bezzina C.R., Biagini E., Blom N.A., de Boer R.A. (2023). 2023 ESC Guidelines for the management of cardiomyopathies. Eur. Heart J..

[15] Ommen S.R., Ho C.Y., Asif I.M., Balaji S., Burke M.A., Day S.M., Dearani J.A., Epps K.C., Evanovich L., Ferrari V.A. (2024). 2024 AHA/ACC/AMSSM/HRS/PACES/SCMR guideline for the management of hypertrophic cardiomyopathy: A report of the American Heart Association/American College of Cardiology Joint Committee on Clinical Practice Guidelines. J. Am. Coll. Cardiol..

[16] Liebregts M., Faber L., Jensen M.K., Vriesendorp P.A., Hansen P.R., Seggewiss H., Horstkotte D., Adlova R., Michels M., Bundgaard H. (2018). Validation of the HCM Risk-SCD model in patients with hypertrophic cardiomyopathy following alcohol septal ablation. Europace.

[17] Ruiz-Salas A., García-Pinilla J.M., Cabrera-Bueno F., Fernández-Pastor J., Peña-Hernández J., Medina-Palomo C., Barrera-Cordero A., De Teresa E., Alzueta J. (2016). Comparison of the new risk prediction model (HCM Risk-SCD) and classic risk factors for sudden death in patients with hypertrophic cardiomyopathy and defibrillator. Europace.

[18] Fernández A., Quiroga A., Ochoa J.P., Mysuta M., Casabé J.H., Biagetti M., Guevara E., Favaloro L.E., Fava A.M., Galizio N. (2016). Validation of the 2014 European Society of Cardiology Sudden Cardiac Death Risk Prediction Model in Hypertrophic Cardiomyopathy in a Reference Center in South America. Am. J. Cardiol..

[19] Maron B.J., Casey S.A., Chan R.H., Garberich R.F., Rowin E.J., Maron M.S. (2015). Independent Assessment of the European Society of Cardiology Sudden Death Risk Model for Hypertrophic Cardiomyopathy. Am. J. Cardiol..

[20] O’Mahony C., Jichi F., Ommen S.R., Christiaans I., Arbustini E., Garcia-Pavia P., Cecchi F., Olivotto I., Kitaoka H., Gotsman I. (2018). International External Validation Study of the 2014 European Society of Cardiology Guidelines on Sudden Cardiac Death Prevention in Hypertrophic Cardiomyopathy (EVIDENCE-HCM). Circulation.

[21] Choi Y.J., Kim H.K., Lee S.C., Park J.B., Moon I., Park J., Kim Y.J., Sohn D.W., Ommen S. (2019). Validation of the hypertrophic cardiomyopathy risk-sudden cardiac death calculator in Asians. Heart.

[22] Nakano M., Kondo Y., Nakano M., Kajiyama T., Miyazawa K., Hayashi T., Ito R., Takahira H., Kobayashi Y. (2021). Predicting therapies in Japanese hypertrophic cardiomyopathy patients with an implantable cardioverter-defibrillator using the 2014 European Society of Cardiology guidelines. Heart Vessel..

[23] Liu J., Wu G., Zhang C., Ruan J., Wang D., Zhang M., Wang L., Yang Y., Li X., Wang Y. (2020). Improvement in sudden cardiac death risk prediction by the enhanced American College of Cardiology/American Heart Association strategy in Chinese patients with hypertrophic cardiomyopathy. Heart Rhythm.

[24] Dong Y., Yang W., Chen C., Ji J., Zheng W., Zhang F., Yang B., Li X., Zhou X. (2021). Validation of the 2020 AHA/ACC risk stratification for sudden cardiac death in Chinese patients with hypertrophic cardiomyopathy. Front. Cardiovasc. Med..

[25] Qi W., Pu L., Zhang J., Chen H., Tang Z., Wang J., Han Y., Chen Y. (2023). Validation of the risk stratification for sudden cardiac death in Chinese patients with hypertrophic cardiomyopathy. Curr. Probl. Cardiol..

[26] Ma H., Xu F., Liu L., Pan C., Luo R., Liu M., Liu T., Shu Y., Li X. (2025). Age and Sex Differences in the Risk of Sudden Cardiac Death in Patients with Hypertrophic Cardiomyopathy: A Multi-Centre Cohort Study. Vasc. Health Risk Manag..

[27] Chan R.H., Maron B.J., Olivotto I., Pencina M.J., Assenza G.E., Haas T., Lesser J.R., Gruner C., Crean A.M., Rakowski H. (2014). Prognostic value of quantitative contrast-enhanced cardiovascular magnetic resonance for the evaluation of sudden death risk in patients with hypertrophic cardiomyopathy. Circulation.

[28] Zhao K., Zhu Y., Chen X., Yang S., Yan W., Yang K., Song Y., Cui C., Xu X., Zhu Q. (2024). Machine learning in hypertrophic cardiomyopathy: Nonlinear model from clinical and CMR features predicting cardiovascular events. Cardiovasc. Imaging.

[29] Chan R.H., van der Wal L., Liberato G., Rowin E., Soslow J., Maskatia S., Chan S., Shah A., Fogel M., Hernandez L. (2024). Myocardial Scarring and Sudden Cardiac Death in Young Patients with Hypertrophic Cardiomyopathy: A Multicenter Cohort Study. JAMA Cardiol..

